# Anatomical and phylogenetic investigation of the genera *Alabastrina* Kobelt, 1904, *Siretia* Pallary, 1926, and *Otala* Schumacher, 1817 (Stylommatophora, Helicidae)

**DOI:** 10.3897/zookeys.843.32867

**Published:** 2019-05-09

**Authors:** Jeannette Kneubühler, Rainer Hutterer, Beat Pfarrer, Eike Neubert

**Affiliations:** 1 Naturhistorisches Museum der Burgergemeinde Bern, 3005 Bern, Switzerland; 2 Institute of Ecology and Evolution, University of Bern, 3012 Bern, Switzerland; 3 Zoologisches Forschungsmuseum Alexander Koenig, 53113 Bonn, Germany

**Keywords:** *
Alabastrina
*, genital anatomy, integrative taxonomy, Morocco, *
Otala
*, phylogeny, *
Siretia
*, Spain

## Abstract

This study presents new insights in the anatomy of genital organs of some large helicid gastropods from northern Africa. The genetic analysis with the markers COI, 16S, H3, and 5.8 S rRNA+ITS2 reveales a high support for *Alabastrina* and *Otala* as separate evolutionary lineages within the Otalini. The position of *Siretia* as another separate lineage within the Otalini is discussed. “*Tingitanaminetteidecussata*” clusters within the *O.xanthodon* clade and confirms that the genus *Tingitana* can be synonymised with *Otala*. The genus *Alabastrina* differs from all other known genera by possession of a penial appendix. This character state is also found in topotypic *A.tistutensis*. Examination of the twin penial papilla system in *Otala* recovers a reduction of the proximal penial papilla in *O.punctata*. The position of *Helixmurcica* as a separate subspecies of *O.lactea* is not supported, and it is here considered to be a synonym of the latter species.

## Introduction

Working with the terrestrial molluscs from northern Africa, students are faced with a confusing situation: an enormous number of species- and genus-level taxa are available to arrange the malacodiversity but for many groups a modern treatment is missing. As a result, this important part of the Palaearctic fauna is still in a chaotic state (Rour et al. 2002). The major problem in the Helicidae is the absence of a stable generic concept that is based on recognisable character states. This can be morphological, anatomical, or genetic data. For this reason, we follow the idea of integrative taxonomy and try to draw conclusions based on a synopsis of these types of traits.

Research on the malacofauna of northern Africa was mainly elaborated by three researchers, Bourguignat (1829–1892), Kobelt (1840–1916), and Pallary (1869–1942), who laid a fundament so strict that it is followed more or less until today. This system was more or less supported by P Hesse (1911) by his anatomical research on some groups of Helicidae. His research was the onset of the valorisation of genital morphology as another source of characters and character states. Amongst others, he investigated species, which are treated also in this publication. Unfortunately, Hesse restricted his research to the outer morphology of the genital organs thus missing the highly informative traits found in the lumen. While in the remaining part of the western Palaearctic, taxonomy of terrestrial snails went through a phase of deep changes, northern Africa was left more or less untouched. This situation is currently changing, and several papers were published in the last years which resulted in new data, for example on the Helicidae (Psonis et al. 2013, Neubert 2014, Neubert and Korábek 2015, Walther et al. 2016, Bouaziz-Yahiatene et al. 2017). Recently, Holyoak and Holyoak (2017) published a major paper on the large group of Otalini G Pfeffer, 1930, which has its centre of radiation in the north-west of Africa. In this paper, the authors went through numerous available names and came up with a radical solution following a lumping approach.

The investigation in this study is mainly based on specimens collected by the second author during his excavation campaigns in north-eastern Morocco (Hutterer et al. 2011a, b. 2014). The taxonomic investigation of terrestrial molluscs was part of an archaeological study of various cave sediments in the Rif region (Mikdad et al. 2000).

This study aims to serve as an addition to the recent studies on helicid phylogeny. Due to the restricted number of taxa available in our study, we here can add only some remarks to the ongoing work on the north African Helicidae. Particular emphasis is laid on filling gaps in the knowledge of the anatomy of the genital organs. It has to be stressed that the investigation of this complex of organs should always include the structure of the internal lumina; they certainly help in identifying autapomorphic character states. In addition, we supply new data on shell and anatomical traits, and present a first genetic approach to some of the genera involved using the following markers: cytochrome c oxidase subunit I (COI), 16S rRNA (16S), histone 3 (H3), and partial sequence of 5.8 S rRNA flanking the internal transcribed spacer 2 (ITS2).

## Material and methods

### Specimens investigated

**Figure 1.**
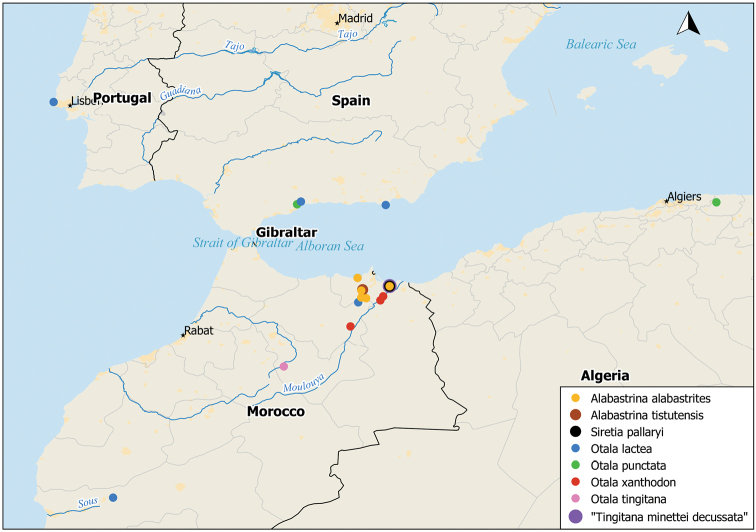
Sampling locations of the investigated specimens. This map was produced with QGIS (2016, v2.18.12) using the Natural Earth data set.

The specimens were collected in Morocco and Algeria between 1998 and 2015. Reference specimens from Spain and Portugal could be included. Detailed sampling locations of the investigated specimens are given in Fig. 1 and Table 1. The voucher number and the GenBank accession numbers for the obtained DNA sequences can be found in Table 1. All specimens used in this study are housed in the Natural History Museum Bern, Switzerland.

**Table 1.** Detailed list of the sampling sites and the GenBank accession numbers of the investigated specimens.

Species	Locality	Latitude	Longitude	Voucher	GenBank accession number COI	GenBank accession number 16S	GenBank accession number H3	GenBank accession number ITS2
* Alabastrina alabastrites *	Morocco, Montes de Kebdana, Kebdana Mountain/ Rif	35.027N, 2.614W		NMBE-549817	MK754458	MK585087	MK728781	MK585111
	Morocco, Rif Jbel Fiztoutine w Hills El Batel	34.938N, 3.193W		NMBE-549813	MK754457	MK585086	MK728780	MK585110
	Morocco, Cave Ifri n’Ammar, 20 km SW Berkane	34.782N, 3.094W		NMBE-549812	MK754456	MK585085	MK728779	MK585109
	Morocco, Hassi Ouenzga nach Afso/ Oriental	34.796N, 3.195W		NMBE-549811	MK754455	MK585084	MK728778	MK585108
	Morocco, Etsedda/ Kebdane	35.195N, 3.269W		NMBE-549816	MK754459	MK585088	MK728782	MK585112
* Alabastrina tistutensis *	Morocco, Rif, Tiztoutine, village bouaza	34.955N, 3.166W		NMBE-555174	MK754469	MK585099	MK728792	MK585123
* Allognathus balearicus *	Spain, Mallorca, Escorça	39.822N, 2.887E		EHUMC-1051	KR705026	KR704986	no data	no data
* Arianta arbustorum *	Austria, Upper Austria, Höllengebirge Mts	no data	no data	NHM-109000	KF596871	KF596823	KF596915	no data
* Helix melanostoma *	Tunisia, Kasserine	35.172N, 8.831E		NMBE-540550	MF564162	MF564116	MF564178	no data
	France, between Rabieux and Saint-Félix-de-Lodez/ Herault	43.663N, 3.441E		NMBE-520822	MK754471	MF564115	MF564177	no data
* Marmorana muralis *	Italy, Rome	41.885N, 12.481E		MN-2554	KR705023	KR704983	no data	no data
* Massylaea constantina *	Algeria, Ighil Bourmi	36.487N, 4.061E		NMBE-540545	MF564168	MF564122	MF564185	no data
* Massylaea vermiculata *	Algeria, Makouda, Tizi Ouzou/ Kabylie	36.791N, 4.066E		NMBE-540544	MF564159	MF564112	MF564174	no data
* Otala lactea *	Spain, Finca de la Concepción, N Málaga	36.760N, 4.428W		NMBE-554174	MK754463	MK585093	MK728786	MK585117
	Spain, Punta Entinas, W Almería	36.690N, 2.694W		NMBE-554175	MK754464	MK585094	MK728787	MK585118
	Spain, Punta Entinas, W Almería	36.690N, 2.694W		NMBE-554176	MK754465	MK585095	MK728788	MK585119
	Portugal, W Almocageme/ Sintra Cascais National Park	38.798N, 9.485W		NMBE-553246	MK754460	MK585089	MK728783	MK585113
	Morocco, Hassi Ouenzga/ Oriental	34.698N, 3.256W		NMBE-555171	MK754452	MK585081	MK728775	MK585105
	Morocco, Hassi Ouenzga/ Oriental	34.698N, 3.256W		NMBE-549814	MK754468	MK585098	MK728791	MK585122
	Morocco, West of Aoulouz/ Souss-Massa-Draa	30.709N, 8.268W		NMBE-549951	MK754472	MK603015	MK728794	MK602877
	Morocco, Etsedda/ Kebdane	35.195N, 3.269W		NMBE-545594	MK754448	MK585077	MK728771	MK585101
* Otala punctata *	Spain, El Tarajal, W Málaga	36.705N, 4.506W		NMBE-554171	MK754462	MK585092	MK728785	MK585116
	Spain, El Tarajal, W Málaga	36.705N, 4.506W		NMBE-554172	MK754467	MK585097	MK728790	MK585121
	Algeria, Makouda, Tizi Ouzou/ Kabylie	36.745N, 4.068E		NMBE-534228	MK754466	MK585096	MK728789	MK585120
* Otala tingitana *	Morocco, Tarzout de Guigou/ Boulmane, NW Boulmane	33.381N, 4.778E		NMBE-510549	no data	no data	no data	no data
* Otala xanthodon *	Morocco, Kebdana, Moulouya valley S Mechraa Elmalh	34.821N, 2.745W		NMBE-555169	MK754450	MK585079	MK728773	MK585103
	Morocco, Kebdana, Moulouya valley S Mechraa Elmalh	34.821N, 2.745W		NMBE-555170	MK754451	MK585080	MK728774	MK585104
	Kebdana, Moulouya valley below barrage	34.739N, 2.803W		NMBE-549825	MK754453	MK585082	MK728776	MK585106
	Kebdana, Moulouya valley below barrage	34.739N, 2.803W		NMBE-549826	MK754454	MK585083	MK728777	MK585107
	Morocco, Montes de Kebdana, Kebdana Mountain/ Rif	35.027N, 2.614W		NMBE-549841	MK754473	MK603016	MK728795	MK602878
	Morocco, Montes de Kebdana, Djebel Sebaa Reyal/ Rif	35.030N, 2.613W		NMBE-549843	MK754474	MK603017	MK728796	MK602879
	Morocco, Guercif, Oued Melloulon/ Taza al-Hoceima	34.207N, 3.414W		NMBE-549820	MK754449	MK585078	MK728772	MK585102
* Siretia pallaryi *	Morocco, Montes de Kebdana, Kebdana Mountain/ Rif	35.027N, 2.614W		NMBE-549815	MK754461	MK585090	MK728784	MK585114
* Theba subdentata subdentata *	Morocco, West of Aoulouz/ Souss-Massa-Draa	30.709N, 8.268W		NMBE-549949	MF564172	MF564126	MF564188	no data
“*Tingitanaminetteidecussata*”	Morocco, Montes de Kebdana, Djebel Sebaa Reyal/ Rif	35.030N, 2.613W		NMBE-549840	MK754470	MK585100	MK728793	MK585124

Abbreviations of institution:

**MHNL** Musée de Confluence, Lyon

**MNHN** Museum National d’Histoire Naturelle, Paris

**NMBE** Naturhistorisches Museum, Bern

**SMF** Research Institute Senckenberg, Frankfurt

### Molecular study

For total DNA extraction the Qiagen Blood and Tissue Kit (Qiagen; Hilden, Germany) was used in combination with a QIAcube extraction robot. Ca. 0.5 cm^3^ of foot tissue was cut from the foot muscle and placed in a mix of 180 µl ATL buffer and 20 µl Proteinase K. It was then incubated for ca. 4 hours at 56 °C in a heater (Labnet, Vortemp 56, witec AG, Littau, Switzerland). For subsequent DNA extraction the QIAcube extraction robot with the Protocol 430 (DNeasy Blood Tissue and Rodent tails Standard) was used. In this study, two mitochondrial markers (COI and 16S) and two nuclear markers (H3 and 5.8 S rRNA+ITS2) were investigated. PCR mixtures consisted of 12.5 µl GoTaq G2 HotStart Green Master Mix (Promega M7423), 8.5 µl ddH_2_O, 1 µl forward and reverse primer each, and 2 µl DNA template. In Table 2 the used primer pairs for the PCR are listed. Following PCR cycles were used: for COI 2 min at 94 °C, followed by 35 cycles of 1 min at 95 °C, 1 min at 40 °C and 1 min at 72 °C and finally, 5 min at 72 °C; for 16S 5 min at 95 °C, followed by 45 cycles of 30 s at 95 °C, 30 s at 48 °C and 45 s at 72 °C, and finally, 5 min at 72 °C; for H3 3 min at 95 °C, followed 40 cycles of 1 min at 95 °C, 1 min at 42 °C and 1 min at 72 °C, and finally, 10 min at 72 °C, and for 5.8 S rRNA+ITS2 1 min at 96 °C, followed by 45 cycles of 30 s at 94 °C, 30 s at 50 °C and 1 min at 72 °C, and finally, 10 min at 72 °C (SensoQuest Tabcyclet and Techne TC-512, witec AG, Littau, Switzerland). The purification and sequencing of the PCR product was performed by LGC (LGC Genomics Berlin, Germany). Interpretation of Bootstrap values: 70 to 80 = moderate support; 80 to 90 = well supported; > 90 = high support. Bayesian posterior probabilities: values above 0.95 are significant support.

**Table 2.** Used primer pairs for the two mitochondrial and two nuclear markers.

Gene	Primer	Sequence	Sequence length (bp)	Reference
COI	LCO1490	5’-GGTCAACAAATCATAAAGATATTGG-3’	680	Folmer et al. 1994
	HCO2198	5’-TAAACTTCAGGGTGACCAAAAAATCA-3’		
16S	16S ar	5’-CGC CTG TTT ATC AAA AAC AT-3’	440	Simon et al. 1994
	16S br	5’- CCG GTC TGA ACT CTG ATC AT -3’		
H3	H3AD	5’-ATGGCTCGTACCAAGCAGACVGC-3’	380	Colgan et al. 1998
	H3BD	5’-ATATCCTTRGGCATRATRGTGAC-3’		
ITS2	ITS2ModA	5’-GCTTGCGGAGAATTAATGTGAA-3’	900	Bouaziz-Yahiatene et al. 2017
	ITS2ModB	5’-GGTACCTTGTTCGCTATCGGA-3’		

### Phylogenetic analyses

For the phylogenetic analyses sequences obtained from GenBank were included as outgroups: *Ariantaarbustorum* (Linnaeus, 1758) (Cadahia et al. 2014), *Marmoranamuralis* (OF Müller, 1774), and *Allognathusbalearicus* (Rossmässler, 1838) (= *Allognathushispanicus* (Rossmässler, 1838)) (Neiber and Hausdorf 2015). Additionally, sequences of *Helixmelanostoma* Draparnaud, 1801, *Thebasubdentatasubdentata* (Férussac, 1821), *Massylaeaconstantina* (E Forbes, 1838) and *Massylaeavermiculata* (OF Müller, 1774) from the study of Bouaziz-Yahiatene et al. 2017 were also included as outgroups. These species were selected to identify the phylogenetic placement of the focal taxa investigated in this study.

For sequence processing and editing the software package Geneious v9.1.8 (Biomatters Ltd) was used. The protein-coding gene fragments of COI and H3 were defined in two data blocks. The first two codon positions were defined as one block and the third codon position as a second block. The non-coding regions from 16S and 5.8 S rRNA+ITS2 were defined as a single data block. Partitionfinder-2.1.1 (Lanfear et al. 2012) was applied for searching optimal evolutionary models for the partitions using the corrected Akaike Information Criterion (cAIC). RAxML plug-in for Geneious (Stamatakis 2006) was implemented for computing ML inference, using Geneious’ plug-in with rapid bootstrapping setting, the search for the best scoring ML tree and 1500 bootstrapping replicates. Bayesian Inference (BI) was performed using Mr. Bayes v3.2.6 ×64 (Huelsenbeck and Ronquist 2001; Ronquist and Huelsenbeck 2003; Altekar et al. 2004) through the HPC cluster from the University of Bern (http://www.id.unibe.ch/hpc). For the concatenated data set, Partitionfinder-2.1.1 was used for finding the optimal evolutionary models for each subset with the model = all function. The Monte Carlo Markov Chain (MCMC) parameter was set as follows: starting with four chains and four separate runs for 20 million generations with a tree sampling frequency of 1000 and a burn in of 25%.

### Anatomical and morphological study

Living animals were killed in boiling water and stored for one day in 80% ethanol. The next day, the ethanol was exchanged and the specimens were stored in the fridge at 5 °C until DNA extraction and dissection. Our experience showed that this procedure maintains the soft tissue and is essential for proper anatomical studies, as well as for the conservation of DNA. The dissection of the snail genitalia took place under a stereomicroscope (Leica MZ12) using thin tweezers and scissors. The genitalia were dissected from the body, spread on a wax bedded bowl, and properly pinned with small needles. The total length of the situs was measured using a calliper (Mitutoyo). Proportions between different parts of the genitalia were estimated using the total situs length as a reference. Additionally, the inner structures of the penis and the epiphallus were investigated. Pictures of the situs were taken with a Leica DFC425 microscope camera using an image-processing program (IMS Client V15Q4, Imagic, Switzerland). The empty shells were imaged using a camera (Canon EOS 50D) in a frontal, lateral, apical, and ventral position. The shell height and shell diameter were measured with perpendicular shell axis with the calliper.

Abbreviations used in the anatomical descriptions and figures:

**At** atrium

**AG** albumin gland

**AS** atrial stimulator

**BC** bursa copulatrix

**BCD** diverticulum of the bursa copulatrix

**D** shell diameter

**DS** dart sac

**Ep** epiphallus

**Fl** flagellum

**FO** free oviduct

**H** shell height

**HD** hermaphroditic duct

**MG** mucus glands

**MRP** musculus retractor penis

**PA** penial appendix

**Pe** penis

**PF** penial flap

**PP1** proximal penial papilla

**PP2** distal penial papilla

**PS** penis sheath

**Va** vagina

**VD** vas deferens

## Results

### Phylogenetic results

**Figure 2.**
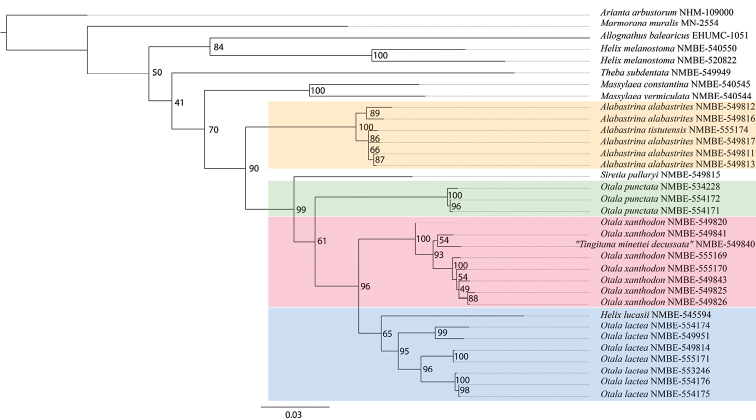
Maximum Likelihood (RAxML) tree based on concatenated data set of COI, 16S, H3, and 5.8 S rRNA+ITS2. Numbers represent bootstrap support values from the ML analysis.

**Figure 3.**
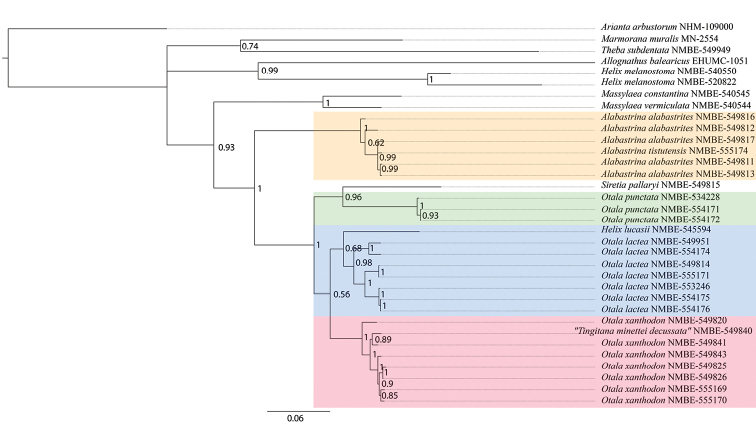
Bayesian Inference tree based on concatenated data set of COI, 16S, H3, and 5.8 S rRNA+ITS2. Numbers represent Bayesian posterior probabilities.

The RAxML analysis of the concatenated data set (Fig. 2) recovered the genus *Alabastrina* as sister genus to *Siretia* and *Otala*. This node is supported with a ML support value of 90. The species *A.tistutensis* Galindo, 2018 clusters within the five specimens of *A.alabastrites* (Michaud, 1833). The monophyly of *S.pallaryi* (Kobelt, 1909) and *Otala* (and thus the separation of *S.pallaryi* and *Alabastrina*) is highly supported (bootstrap value of 99). The monophyly of *Otala* is not statistically supported (bootstrap value of 61). Within *Otala* we recovered three major clades, i.e., *O.punctata* (OF Müller, 1774), *O.lactea* (OF Müller, 1774), and *O.xanthodon* (Anton, 1838). The specimen of “*Tingitanaminetteidecussata*” (nomen nudum) clusters within the *O.xanthodon* clade. The monophyly of *O.lactea* is not statistically supported (bootstrap value of 65). Within *O.xanthodon* there are some nodes with very low support, especially the node which includes “*Tingitanaminetteidecussata*” (NMBE 549840). *Otalal.murcica* (Rossmässler, 1854) (NMBE-554175 and NMBE-554176 in Figs 2, 3) nests within the *O.lactea* clade. Both, the separate mitochondrial and nuclear tree show the same topology as the concatenated tree. They can be found in the supplementary material (Suppl. materials 1, 2).

The Bayesian Inference analysis of the concatenated data set (Fig. 3) recovered the monophyly of *Alabastrina*. This node is statistically supported (posterior probability of 1). The monophyly of *S.pallaryi* and *Otala* and thus the separation of *S.pallaryi* and *Alabastrina* is fully supported. There is no difference in both types of analyses in the *O.lactea* and the *O.xanthodon* clade. The separate mitochondrial and nuclear trees can be found in the supplementary material (Suppl. materials 3, 4).

### Taxonomic accounts

The nomenclature of the parts of the genital organs follows Neubert and Bank (2006) and Neubert (2014). In Table 3, the traits of the genital organs are summarised.

**Table 3.** Traits of genital organs.

	* A. alabastrites *	* A. tistutensis *	* S. pallaryi *	* O. lactea *	* O. punctata *	* O. xanthodon *
relative size of the AS	medium	medium	no data	large	large	large
penial flap	yes	yes	no data	no	no	no
relative size of the Fl	short	short	no data	long	medium	long
relationship BC:BCD	1:1	no data	no data	1.5:2	1:1	1.5:2
no. of penial papillae	1	1	no data	2	1	2
penial appendix	yes	yes	no data	no	no	no

### *Alabastrina* Kobelt, 1904

1904 *Alabastrina* Kobelt, in Rossmässler: Iconographie der Land- & Süsswasser-Mollusken, (2) 11: 33, 132, 194 [type species *Helixalabastrites* Michaud, 1833 by OD].

1904 *Alabastra* Kobelt, in Rossmässler: Iconographie der Land- & Süsswasser-Mollusken, (2) 11: 100.

Currently, this genus is subdivided in six subgenera (Schileyko 2006). This system is more or less completely based on shell characters and only for a few specimens the morphology of the genital organs has been investigated and published. Schileyko (2006: 1794, fig. 2297B, C) shows the genital organs of *Helixhieroglyphicula* Michaud, 1833, which is the type species of *Michaudia* Pallary, 1926 [by original designation]. In his definition of *Alabastrina* sensu lato, he uses the character state “branches of mucus glands before entering common duct form distinct swellings” (Schileyko 2006: 1792). This interesting trait is not seen in any of the *Alabastrina* species investigated by us. Holyoak and Holyoak (2017: 426, Table 1) relegate *Michaudia* into the synonymy of *Otala*, also based on Schileyko’s figure arguing with the conformity in the structure of the interior of the proximal penis. The assumption by Schileyko (2006) that *Alabastrina* agrees with *Otala* on the presence of two penial papillae is wrong.

Without further comment, Holyoak and Holyoak (2017) consider *Loxana* Pallary, 1899 a separate genus, follow Razkin et al. (2015) in leaving Atlasica Pallary, 1917 as a subgenus of Alabastrina, and omit *Lechatelieria* Pallary, 1926. Taxon sampling in Razkin et al. (2015) is not sufficient enough to clearly reveal the subgeneric position of *Atlasica*. Based on our anatomical investigation, the genus *Alabastrina* can now be newly characterised using the following traits of the genital organs: Penis with a single penial papilla (PP) with a central pore, distal penis with penial flap (PF), proximal penis with a small penial appendix (PA); epiphallus and flagellum of similar length; mucus glands (MG) multifid, branches very long and slender.

Nomenclatural remark: Kobelt established the names *Alabastra* and *Alabastrina* simultaneously in the register volume of the “Iconographie”. In this work, he presented a register on the “System der palaearktischen Binnenconchylien”, listing a genus group name together with a single species group name (129 ff.). In the second register (171 ff.), he provided a systematically ordered list with information on all taxa ever published in the “Iconographie”, and affiliated these taxa into the new system as outlined before in register 1. Both registers are accompanied by text dealing with zoogeographic considerations and taxonomic remarks.

The name *Alabastra* was used three times exclusively on page 100 (in combination with a species list). The name *Alabastrina* was used on page 33 (zoogeographic context), page 132 (systematic register combined with the species group name *alabastrites*), page 158 (a list of potential members of *Alabastrina* including *alabastrites*), and finally page 194 (amended list of illustrated taxa of *Alabastrina* sensu Kobelt). According to ICZN 24.2.4 we deem Kobelt to act here as First Reviser, because he consequently used the name *Alabastrina* in his registers. We interpret the name *Alabastra* to constitute an erroneous misspelling.

Both genus group names included species lists of differing composition, the name *alabastrites* was always included (loc. cit.). In the first register, the name *Alabastrina* was combined with a single species (p. 132). We consider this act a designation of the type species by the original author (OD); Schileyko’s note on the type species selection (2006: 1792) as “monotypy” is erroneous.

### *Alabastrinaalabastrites* (Michaud, 1833)

Figs 4–8

1833 *Helixalabastrites* Michaud, Catalogue des testacés vivans envoyés d’Alger par M. Rozet, capitaine au corps royal d’État-Major, au cabinet d’Histoire Naturelle de Strasbourg: 4, figs 6–8 [Oran].

1833 *Helixsoluta* Michaud, Catalogue des testacés vivans envoyés d’Alger par M. Rozet, capitaine au corps royal d’État-Major, au cabinet d’Histoire Naturelle de Strasbourg: 3, figs 9, 10 [Oran].

Type specimens: *Helixalabastrites*: syntype MHNL 45000690; *Helixsoluta*: syntype MHNL 45000679.

Specimens examined: for sequenced specimens, see Table 1.

**Figure 4.**
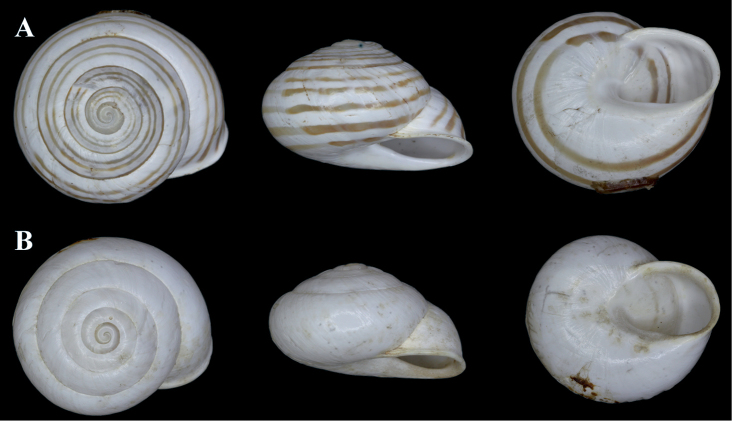
*Alabastrina* type specimens. **A***Helixsoluta*, syntype MHNL 45000679, Oran, Algeria, coll. Michaud, D = 24.15 mm **B***Helixalabastrites*, syntype MHNL 45000690, Oran, Algeria, coll. Michaud, D = 22.48 mm. All photographs by Kneubühler & Neubert, × 1.5.

**Description**. The range of the shell diameter of the investigated specimens is between 14.93–22.77 mm and shell height is between 10.85–13.45 mm. The shell of this species is pale and often with dark brown stripes. Some individuals do not show any stripes at all (Figs 4B, 6A). There is none to one tooth found in the aperture.

**Figure 5.**
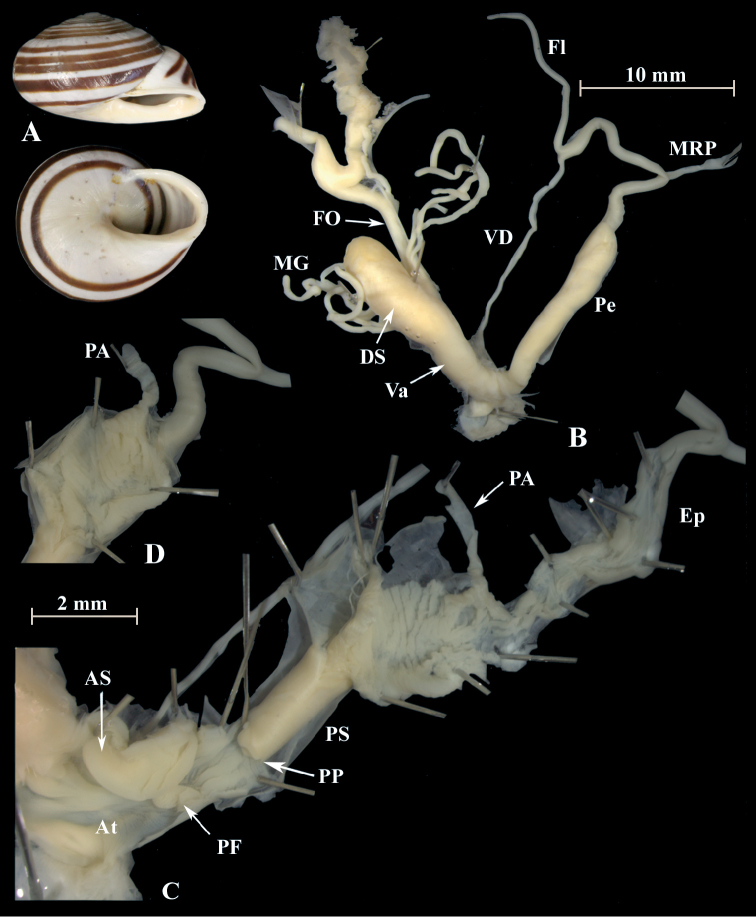
*Alabastrinaalabastrites* (NMBE 549817), Kebdana Mountain, Morocco; **A** shell **B** situs **C** penis **D** penial lumen; D = 21.91 mm, H = 13.36 mm, situs length 27.57 mm (atrium-flagellum). All photographs by Kneubühler, shell × 1.5.

This species has a rather short flagellum which is a bit shorter than the penis. MG are thin and fragile. The epiphallus goes over into the penial lumen without any penial papilla. Parallel but outside of the epiphallus is a penial appendix found. This penial appendix lies next to the epiphallus and is also covered by the penial sheath. It is blind on one side and opens into the penial lumen on the other side (PA in Fig. 5C, D). From there a huge penial papilla (PP) points towards the atrium. The PP is surrounded by massive muscles. In the atrium is a large atrial stimulator found and a smaller is located at the exit of the penis (PF).

**Figure 6.**
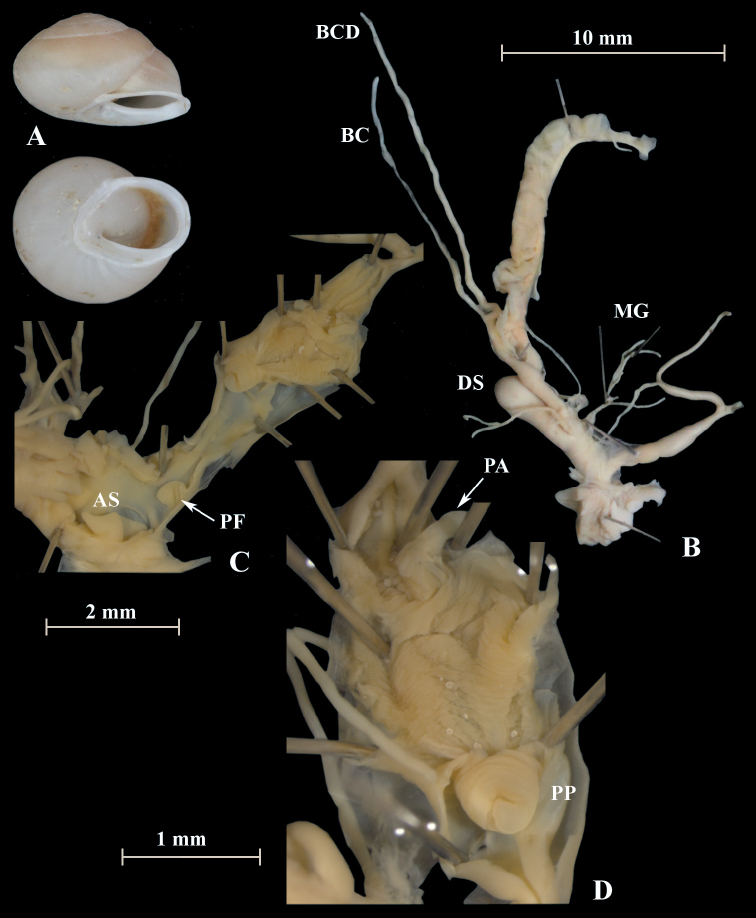
*Alabastrinaalabastrites* (NMBE 549812), cave Ifri n’Ammar, Morocco; **A** shell **B** situs **C** penis **D** penial lumen; D = 19.72 mm, H = 13.00 mm, situs length 26.27 mm (atrium-BCD). BC lost during dissection. All photographs by Kneubühler, shell × 1.5.

**Figure 7.**
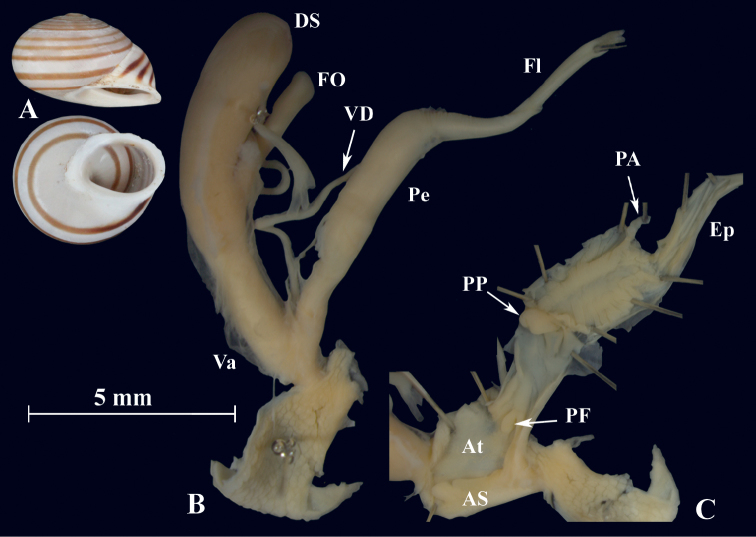
*Alabastrinaalabastrites* (NMBE 549813), hills El Batel, Morocco; **A** shell **B** situs **C** penis; D = 17.25 mm, H = 10.85 mm, situs length 13.46 mm (atrium-flagellum). Situs is not complete. All photographs by Kneubühler, shell × 1.5.

**Figure 8.**
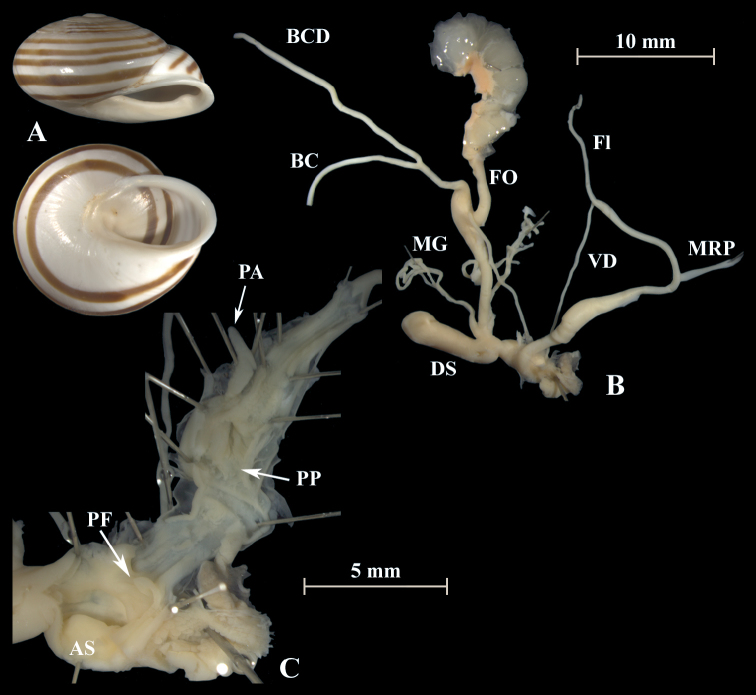
*Alabastrinaalabastrites* (NMBE 549816), Etsedda/ Kebdana, Morocco; **A** shell **B** situs **C** penis; D = 22.77 mm, H = 13.45 mm, situs length 36.84 mm (atrium-BCD). BC destroyed. All photographs by Kneubühler, shell × 1.5.

### *Alabastrinatistutensis* Galindo, 2018

2018 *Alabastrinatistutensis* Galindo, Mostra mondiale, Cupra Marittima (2): 22–26.

Type specimen: *Alabastrinatistutensis*: holotype MMM Cupra Marittima (2): 23.

Specimens examined: for sequenced specimen, see Table 1.

**Figure 9.**
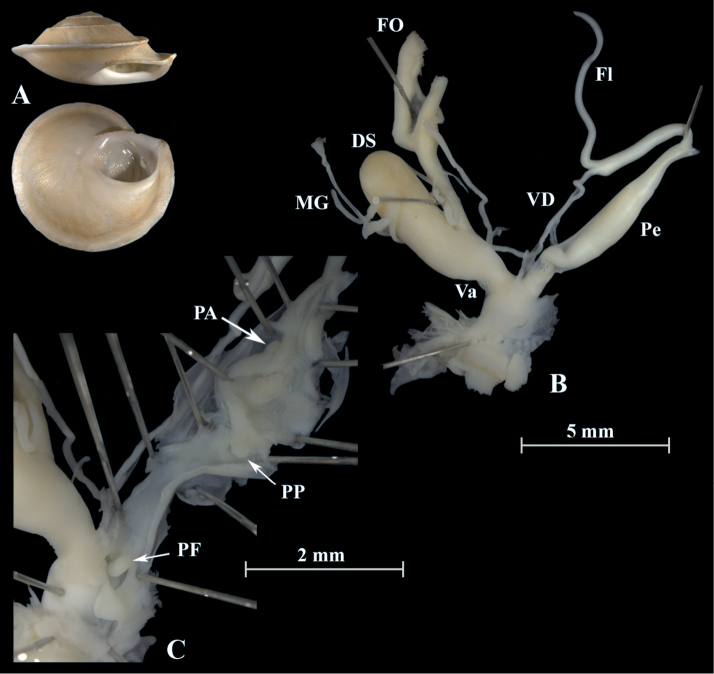
*Alabastrinatistutensis* (NMBE 555174), Tiztoutine, Morocco; **A** shell **B** situs **C** penis; D = 19.59 mm, H = 8.74 mm, situs length 13.51 mm (atrium-flagellum). Situs not complete. All photographs by Kneubühler, shell × 1.5.

**Description**. The shell is pale and characterised by a sharp keel. The aperture is white with a white lip. The mucus glands (MG) are fragile and slender. The flagellum is slightly shorter than the penis. The epiphallus is characterised by longitudinal tissue ridges and goes over into the penial lumen without any penial papilla. Parallel but outside of the epiphallus is a penial appendix found (PA in Fig. 9C). It is together with the epiphallus covered by the penial sheath. The PA is blind on one side and the other side opens into the penial lumen. This species possesses one penial papilla (PP in Fig. 9C) which is slightly smaller than in *A.alabastrites* but it is clearly visible. A large atrial stimulator is found in the atrium and a smaller stimulator is situated in front of the exit of the penis.

### *Siretia* Pallary, 1926

1926 *Siretia* Pallary, Journal de Conchyliologie, 70: 19.

This genus is characterised by a triangular, toothless aperture, the short upper edge of the shell, its flat form, and by having four dark bands (Pallary 1926). Although *Siretia* has a peculiar shell morphology, Schileyko (2006) considers it as a subgenus of *Alabastrina*. Our phylogenetic analyses reveal it as a separate genus.

### *Siretiapallaryi* (Kobelt, 1909)

Figure 10

1909 *Archelixpallaryi* Kobelt, Nachrichtsblatt der Deutschen Malakozoologischen Gesellschaft, 41 (3): 134 [Taforalt im Gebiet der Beni Snassen].

1914 *Archelixpallaryi*, – Kobelt: in Rossmässler: Iconographie der Europäischen Land- & Süsswasser-Mollusken (2) 20: 21, fig. 2790.

1926 *Siretiapallaryi*, Journal de Conchyliologie, 70: 19, figs 5, 6, 8.

Type specimen: *Siretiapallaryi*: syntype SMF 75926.

Specimens examined: for sequenced specimen, see Table 1.

**Figure 10.**
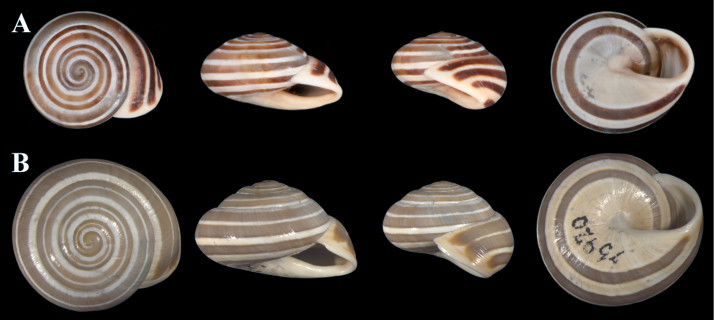
**A***Siretiapallaryi* (NMBE 549815), Kebdana Mountain, Morocco, D = 16.82 mm, H = 8.81 mm; **B***S.pallaryi* (SMF 75926), Teforalt (= Taforalt), Morocco, coll. CR Boettger, D = 19.38 mm. All photographs by Kneubühler & Neubert, × 1.5.

**Description**. In Figure 10B, a syntype of *S.pallaryi* from Teforalt (= Taforalt), Morocco (coll. CR Boettger ex Kobelt) is shown. The type specimen is slightly larger than our investigated specimen (Fig. 10A). Both show similar shell morphology and stripe pattern. Unfortunately, our specimen was badly preserved and a juvenile, therefore no proper investigation of the genital organs could be made.

**Remarks**. Holyoak and Holyoak (2017: 446) attribute this species to A Koch. However, in the description Kobelt explicitly mentions “Koch mss”. Therefore, Kobelt is considered the nomenclatural author of this taxon.

### *Otala* Schumacher, 1817

1817 *Otala* Schumacher, Essai d’un nouveau système des habitations des vers testacés: 58, 191 [type species *Helixlactea* OF Müller, 1774, by subsequent designation Pilsbry, 1895: 323].

1904 Otala (Dupotetia) Kobelt: in Rossmässler: Iconographie der Europäischen Land- & Süsswasser-Mollusken (2) 11: 158 [type species *Helixdupotetiana* Terver, 1839 by original designation].

1918 Alabastrina (Tingitana) Pallary, Bulletin de la Société d’ Histoire naturelle de l’Afrique du Nord, 9 (7): 145 [type species *Archelixminettei* Pallary, 1917 by monotypy].

This genus was recently revised by Holyoak and Holyoak (2017). After examining several hundreds of specimens from Morocco and Algeria, they distinguish five species within the genus *Otala*, i.e., *O.punctata*, *O.lactea*, *O.xanthodon*, *O.tingitana* (Paladilhe, 1875), and *O.hieroglyphicula* (Michaud, 1833). The species formerly attributed to *Tingitana* Pallary, 1918, and *Dupotetia* Kobelt, 1904 (genera which appeared to have species in the area of the Kebdana) are now lumped under *Otalatingitana*. This lumping approach is supported by the molecular study of Helicoidea by Razkin et al. (2015), who revealed that the genus *Tingitana* is nested within *Otala*. In our phylogenetic analysis we included a specimen of the well-known shell form “*Tingitanaminetteidecussata*”, which clustered within the *O.xanthodon* clade thus supporting the results of Razkin et al. (2015) and Holyoak and Holyoak (2017). More taxon sampling is needed to reveal the phylogenetic relationships within *Otala*.

### *Otalalactea* (OF Müller, 1774)

Figs 11–16

Type specimens: *Helixlucasii*: MNHN IM-2000-31721.

Specimens examined: for sequenced specimens, see Table 1.

**Description**. The shell of *O.lactea* is characterized by a dark aperture. The shell diameter of the investigated specimens ranges between 27.01–40.81 mm and shell height between 15.77–21.75 mm. This species has a large and thick penial tube. It has two distinct penial papillae with each a large central pore. The distal penial lumen between the large tongue-shaped atrial stimulator and the distal penial papilla (PP2) exhibits longitudinal ridges. The distal penial papilla is located ca. 2 mm distally to the atrium. The penial chamber which is bordered by the two penial papillae ranges between 2–4 mm and is characterised by strong annular tissue folds. There is a short transformation zone between the proximal penial papilla (PP1) and the epiphallus. The epiphallus is characterised by longitudinal tissue ridges. The flagellum is ca. 1.5× the length of the penis. The BCD is ca. double in length as the BC, except for the specimen in Figure 13, where they are approximately the same length. The vagina is stout and short. The MG consist of two massive stems which subdivide into ten smaller branches.

**Figure 11.**
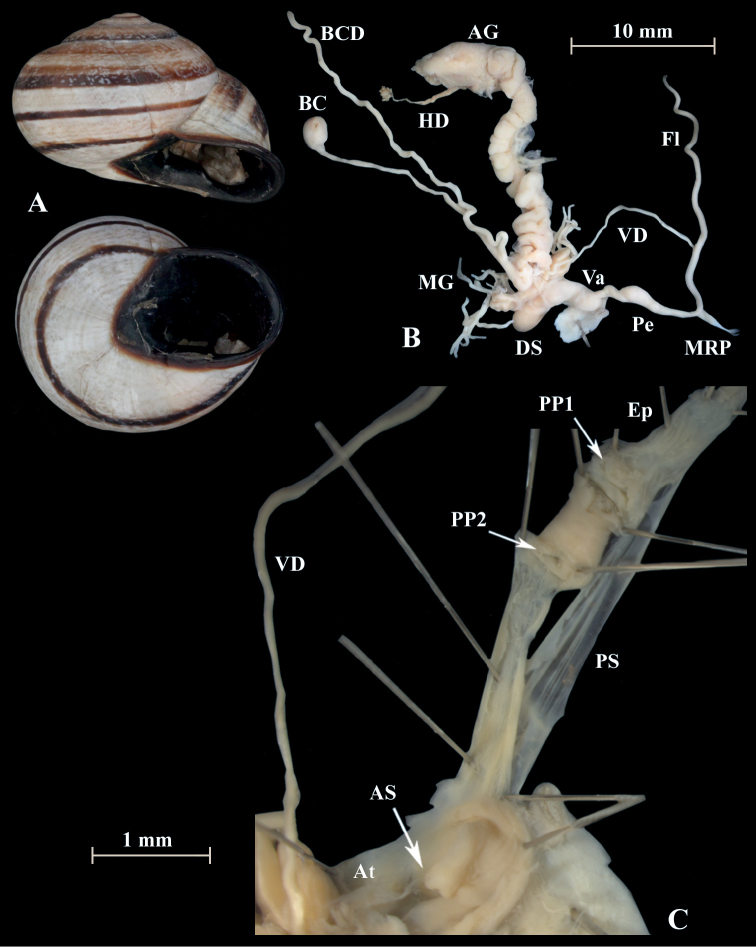
*Otalalactea* (NMBE 553246), W Almocageme, Portugal; **A** shell **B** situs **C** penis and atrium; D = 29.82 mm, H = 18.71 mm, situs length 41.34 mm (atrium-BCD). All photographs by Kneubühler, shell × 1.5.

**Figure 12.**
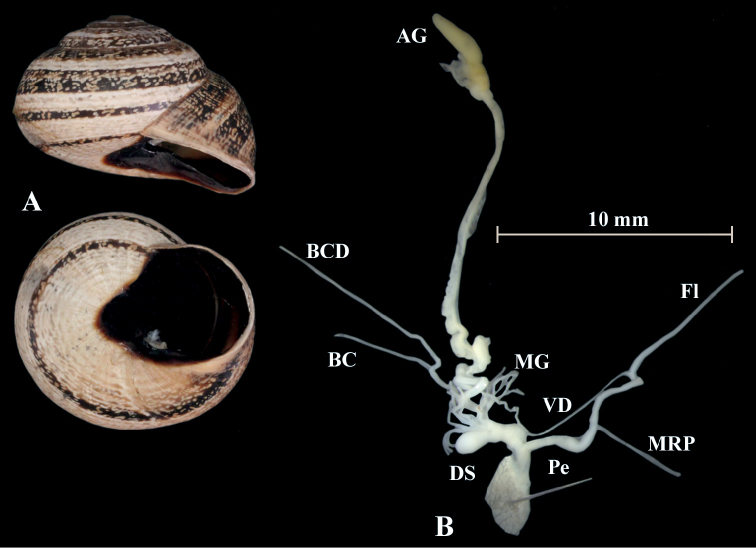
*Otalalactea* (NMBE 554174), N Málaga, Spain; **A** shell **B** situs; D = 27.25 mm, H = 18.60 mm, situs length 22.90 mm (atrium-albumin gland); juvenile, BC destroyed. All photographs by Kneubühler, shell × 1.5.

**Figure 13.**
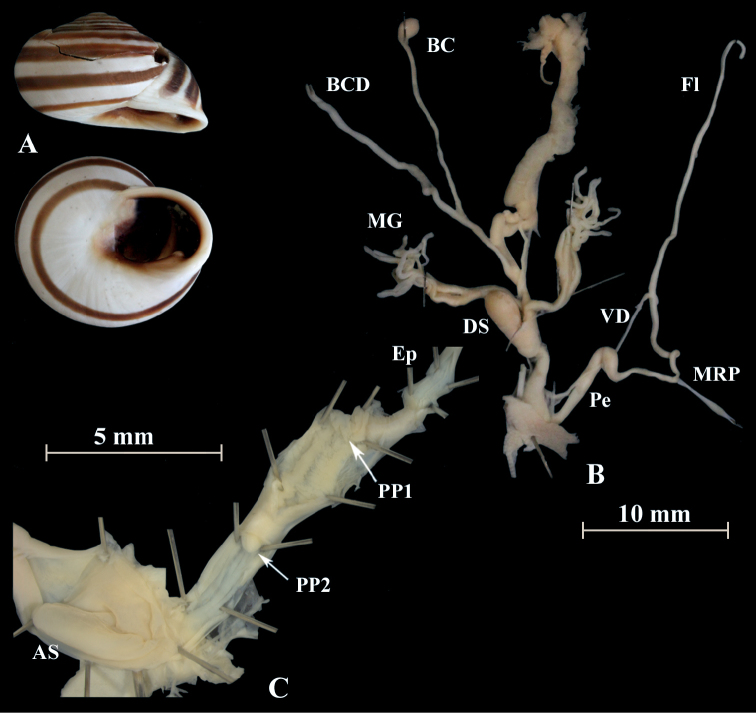
*Otalalactea* (NMBE 555171), Hassi Ouenzga/ Oriental, Morocco; **A** shell **B** situs **C** penis and atrium; D = 22.63 mm, H = 14.85 mm, situs length 34.60 mm (atrium-flagellum). All photographs by Kneubühler, shell × 1.5.

**Figure 14.**
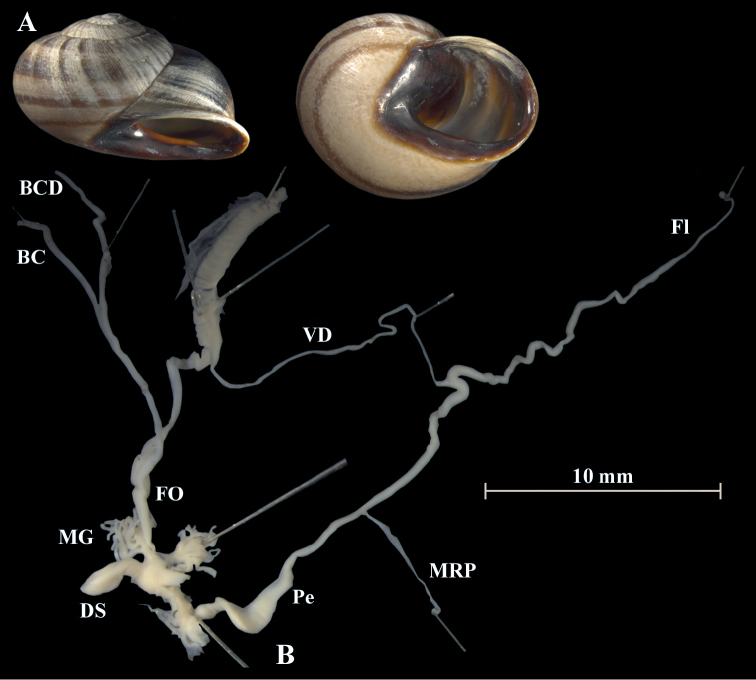
*Otalalactea* (NMBE 549951), W Aoulouz, Morocco; **A** shell **B** situs; D = 27.01 mm, H = 15.77 mm, situs length 30.77 mm (atrium-flagellum); juvenile; situs not complete; BC destroyed. All photographs by Kneubühler, shell × 1.5.

**Figure 15.**
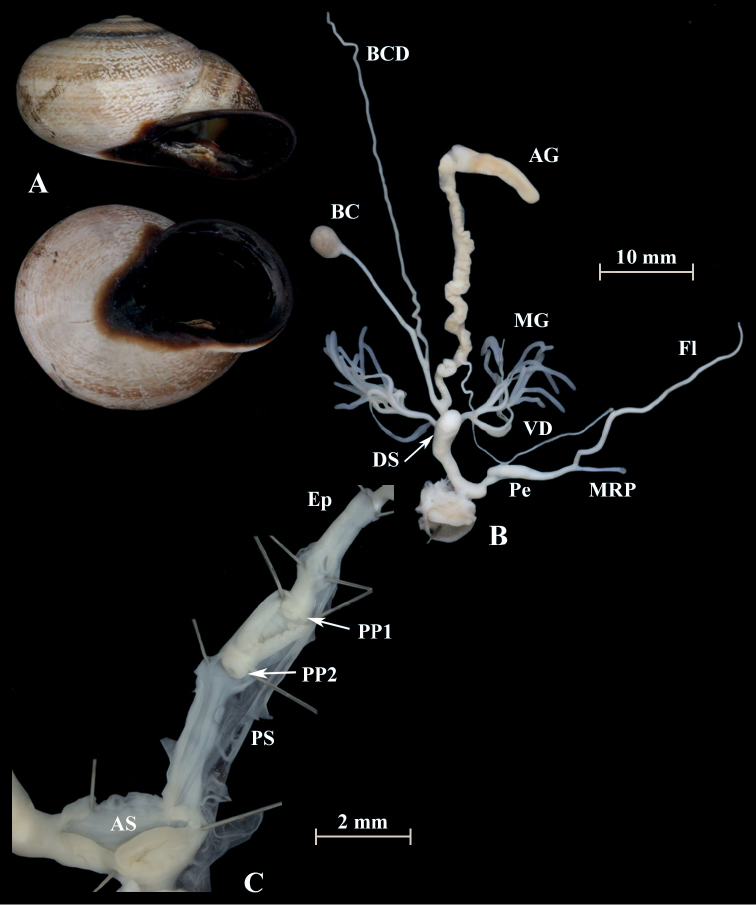
*Otalalactea* (NMBE 554175), W Almería, Spain; **A** shell **B** situs **C** penis and atrium; D = 31.89 mm, H = 18.23 mm, situs length 57.86 mm (atrium-BCD). All photographs by Kneubühler, shell × 1.5.

**Remarks.** The analysis includes also specimens of *O.l.murcica* (Fig. 15) from Almería, Spain, which is the type locality. This taxon is characterised by a larger shell and an aperture, which is enlarged and more reflected (Cadevall and Orozco 2016). The morphology of the genital organs shows no difference to the specimens of *O.lactea* investigated from Portugal or Morocco.

**Figure 16.**
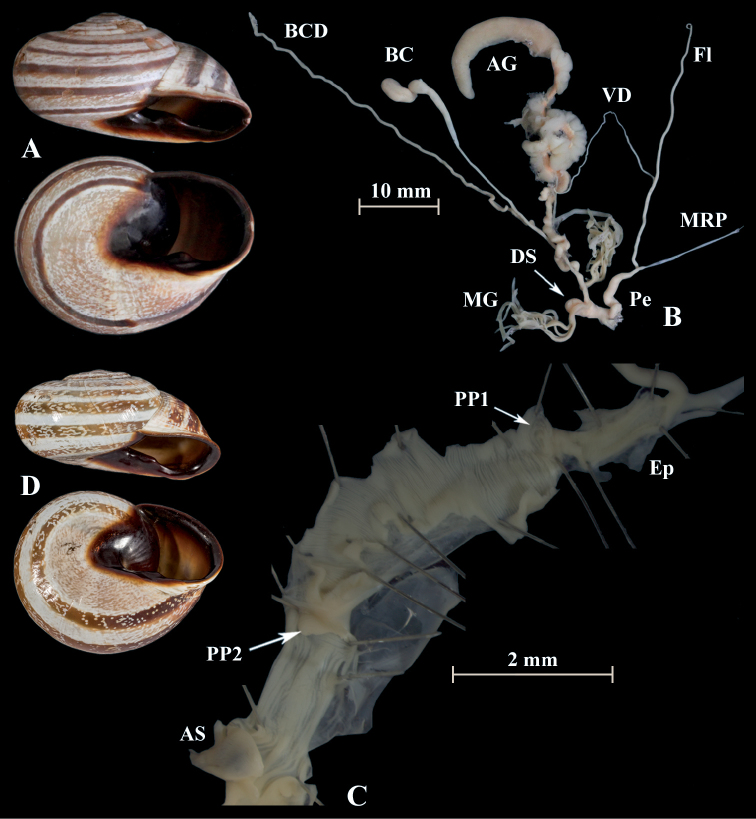
*Otalalactea* (NMBE 545594); Etsedda/Kebdana, Morocco; **A** shell **B** situs **C** penis; D = 40.81 mm, H = 21.75 mm, situs length 61.47 mm (atrium-BCD), BC destroyed; **D***H.lucasii* (syntype MNHN IM-2000-31721), Oran, Algeria, D = 35.4 mm. All photographs by Kneubühler & Neubert, shell original size.

In a small area in north-eastern Morocco, another form of *O.lactea* occurs, namely *Helixlucasii* (Fig. 16D). Our investigation of a specimen from this population revealed some differences in the anatomy of the genital organs (Fig. 16C). The penial chamber is much longer than in the other specimens of *O.lactea*. The length of the penial chamber (PP1-PP2) is 4 mm and the length of the distal penial lumen (PP2-AS) is 1.8 mm. The internal structures differ substantially. Here, the inner walls of this tube are filled by numerous fine transverse ridges arranged in a very dense annular pattern. All other specimens seen so far displayed an irregular network of tissue folds in this section of the penis. Additionally the shell is quite large and flat with a comparatively strong basal tooth or strengthened lip.

### *Otalapunctata* (OF Müller, 1774)

Figs 17, 18

Specimens examined: for sequenced specimens, see Table 1.

**Figure 17.**
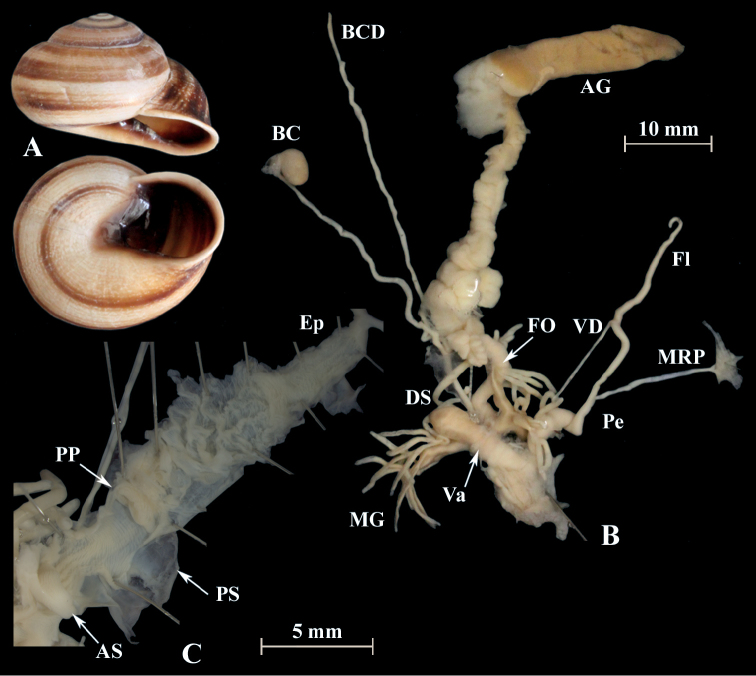
*Otalapunctata* (NMBE 534228); Makouda, Algeria; **A** shell **B** situs **C** penis; D = 36.02 mm, H = 22.37 mm, situs length 59.77 mm (atrium-BCD). All photographs by Kneubühler, shell original size

**Figure 18.**
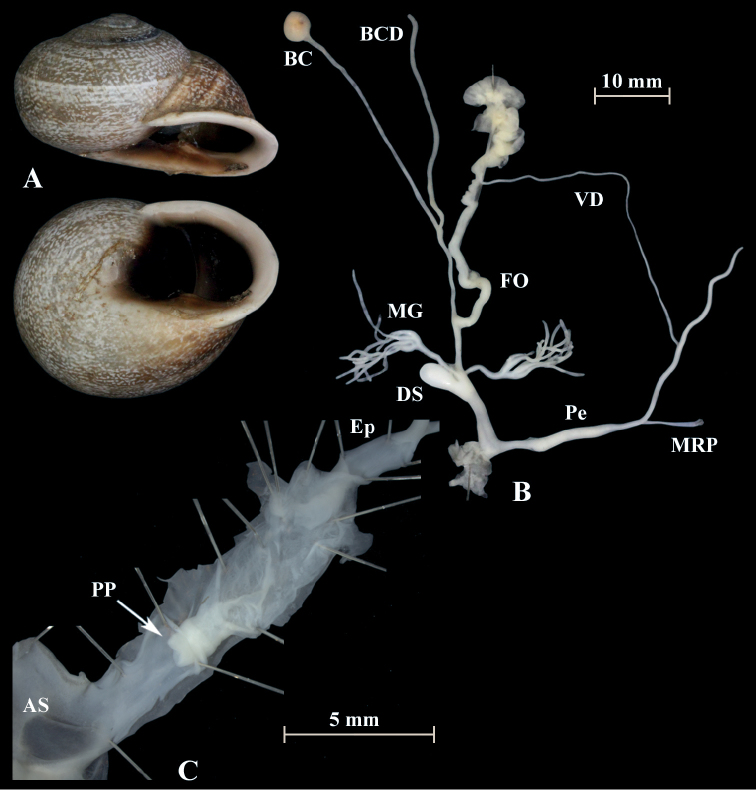
*Otalapunctata* (NMBE 554171); W Málaga, Spain; **A** shell **B** situs **C** penis; D = 30.30 mm, H = 18.05 mm, situs length 70.10 mm (atrium-BC). All photographs by Kneubühler, shell × 1.5.

**Description**. The shell is characterized by a white lip and a basal tooth. This species is characterized by a long and thick penial tube. It has a large penial papilla (PP), which is located ca. 2 mm distally to the atrium (Figs 17, 18) with a large central pore. The second proximal penial papilla is reduced and inconspicuous. The distal penial lumen between the atrial stimulator and the penial papilla exhibits a few low longitudinal ridges intersected by many small annular folds. The proximal lumen between penial papilla and epiphallus is filled by a network of irregularly shaped folds and small and large ridges. The epiphallus is characterised by longitudinal tissue ridges with a small transformation zone at the proximal end of the penial lumen. The flagellum has approximately the same length as the penis. The vagina is short and stout. The mucus glands (MG) consist of two massive stems which subdivide into 10–12 smaller subsequent branches. The BCD has approximately the same length as the BC. They are ca. 3× the length of the flagellum and the penis. The dominant structure in the atrium is a large, folded stimulator, which was also mentioned by De Mattia and Mascia (2011).

### *Otalaxanthodon* (Anton, 1838)

Figs 19–23

Specimens examined: for sequenced specimens, see Table 1.

**Description**. The shell is characterized by a dark aperture with a white and strongly reverted lip. This species possesses one basal tooth. A palatal tooth is found in some specimens. The shell diameters of the investigated specimens range between 21.47–27.77 mm and shell height between 13.37–16.04 mm. *Otalaxanthodon* has two distinct penial papillae with each a large central pore. The distal penial lumen between the atrial stimulator and the distal penial papilla (PP2) exhibits smooth longitudinal tissue ridges. The penial chamber which is bordered by the two penial papillae is filled by a network of irregularly shaped tissue folds and is ca. 3 mm long. There is a short transformation zone between the proximal penial papilla (PP1) and the epiphallus. The epiphallus contains few smooth longitudinal ridges. This species has a large flagellum which is ca. double the length of the penis. The BC is a thin tube and ca. half the length of the BCD. It has two massive mucus glands (MG) which subdivide in four thinner branches of which each again subdivides in two thin branches. The dominant structure in the atrium is a large tongue-shaped stimulator.

**Figure 19.**
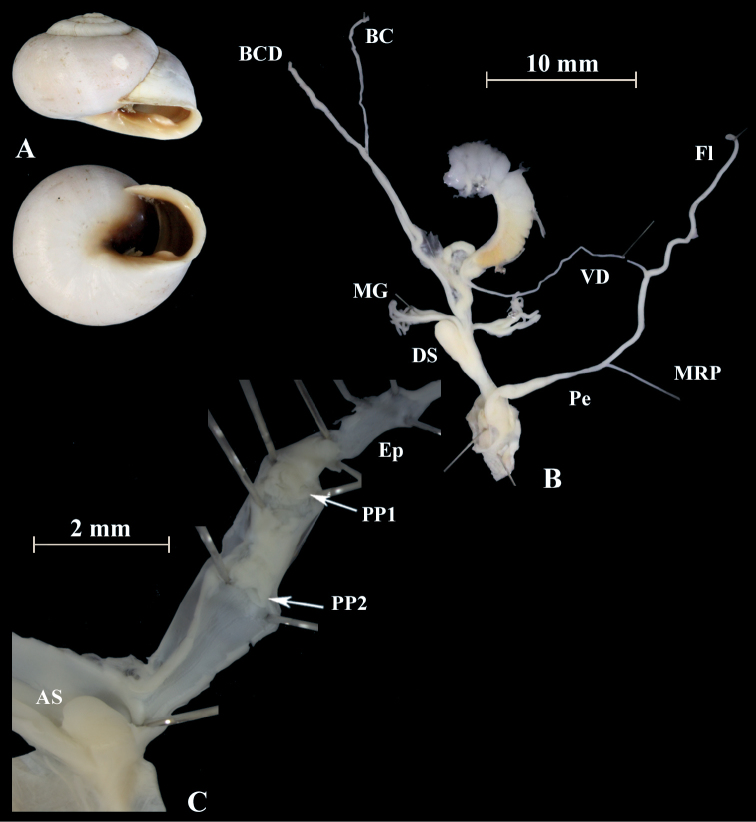
*Otalaxanthodon* (NMBE 549825), Moulouya, Morocco; **A** shell **B** situs **C** atrium and penis; D = 23.13 mm, H = 14.49 mm, situs length 32.68 mm (atrium-BCD). BC destroyed. All photographs by Kneubühler, shell × 1.5.

**Figure 20.**
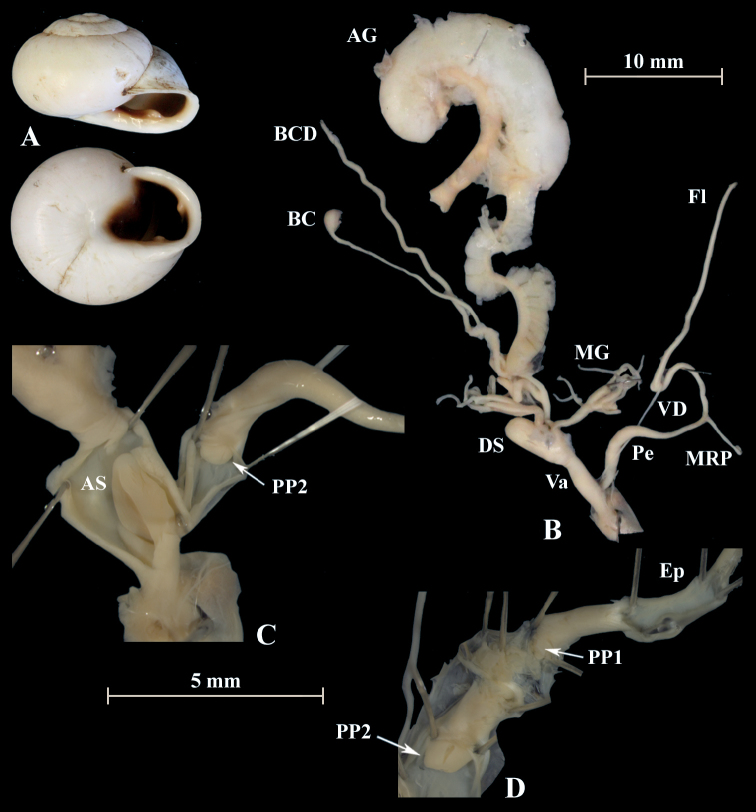
*Otalaxanthodon* (NMBE 549826), Moulouya, Morocco; **A** shell **B** situs **C** atrium and PP2 **D** penial chamber; D = 21.47 mm, H = 14.22 mm, situs length 37.23 mm (atrium-BCD). All photographs by Kneubühler, shell × 1.5.

**Figure 21.**
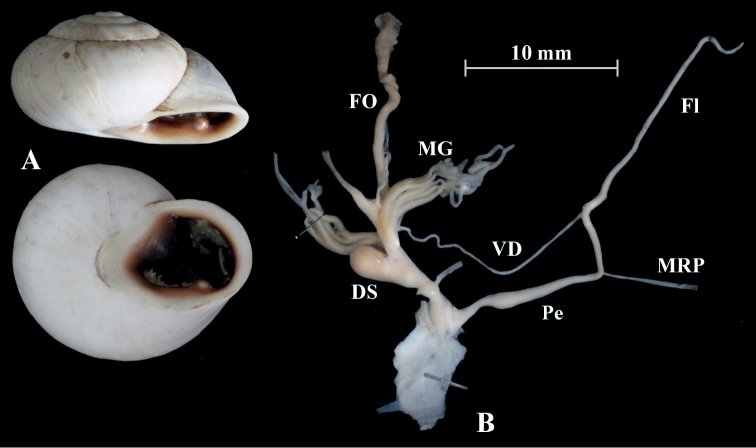
*Otalaxanthodon* (NMBE 549841), Kebdana Mountain, Morocco; **A** shell **B** situs; D = 26.85 mm, H = 15.74 mm, situs length 33.58 mm (atrium-flagellum). Situs not complete. All photographs by Kneubühler, shell × 1.5.

**Figure 22.**
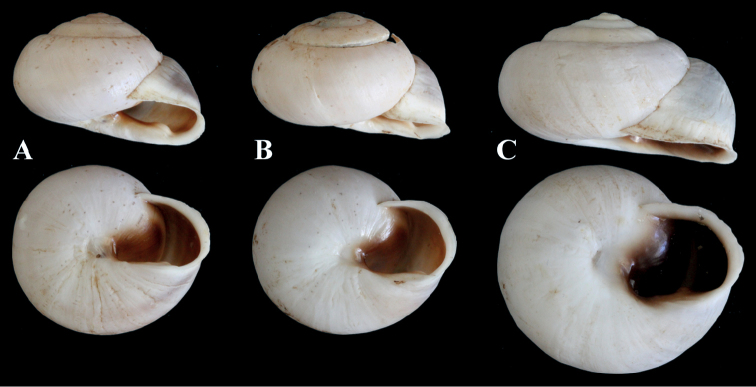
*Otalaxanthodon*, Kebdana, Moulouya valley, Morocco; **A** shell from *O.xanthodon* (NMBE 555169), D = 22.33 mm, H = 13.61 mm; **B** shell from *O.xanthodon* (NMBE 555170), D = 23.10 mm, H = 13.37 mm. Kebdana, Djebel Sebaa Reyal/ Rif **C** shell from *O.xanthodon* (NMBE 549843), D = 27.77 mm, H = 16.04 mm. All photographs by Kneubühler, shell × 1.5.

**Figure 23.**
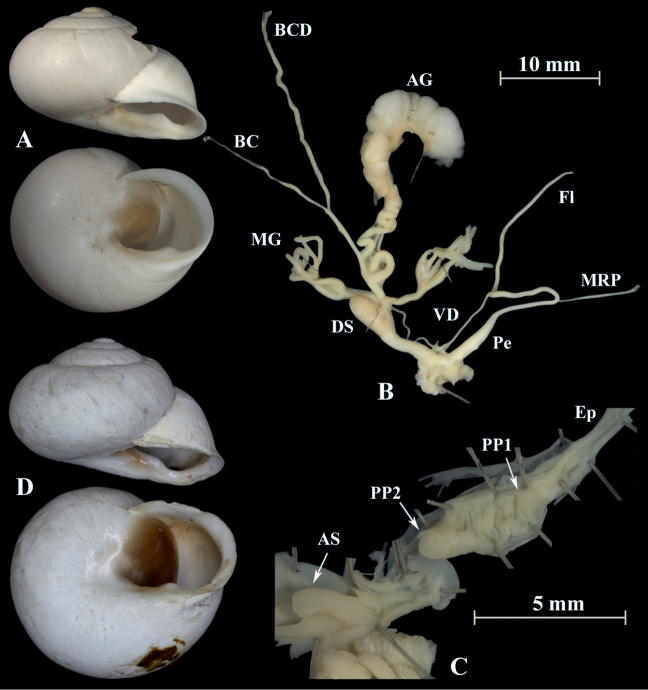
*Otalaxanthodon* (NMBE 549820), Guercif, Morocco; **A** shell **B** situs **C** atrium and penis; D = 26.74 mm, H = 15.96 mm, situs length 42.81 mm (atrium-BCD), BC destroyed; **D***Helixzaffarina* Terver, 1839 (syntype MHNL 45001034), Oran, Algeria, coll. Michaud, D = 29.54 mm. All photographs by Kneubühler & Neubert, shell × 1.5.

### “*Tingitanaminetteidecussata*”

Figs 24, 25

Specimens examined: *Otalatingitana* (NMBE 510549); for the sequenced specimen of “*Tingitanaminetteidecussata*” NMBE 549840, see Table 1.

**Nomenclatorial note**: The name “*decussata* Pallary” is a nomen nudum as already stated by Holyoak and Holyoak (2017: 463). Pallary never made the name available, nor did Llabador (1952). For the latter publication, the provisions of Article 13 ICZN (names published after 1930) rule that every new name must “be accompanied by a description or definition that states in words characters that are purported to differentiate the taxon” or Article 13.1.2. “be accompanied by a bibliographic reference to such a published statement”. No such statements are provided by Llabador. This taxon is well known and often treated as a subspecies of *Tingitanaminettei* (Pallary, 1917) (see for example Cossignani 2014). The genus *Tingitana* Pallary, 1918 is synonymised with *Otala* by Holyoak and Holyoak (2017).

**Description**. The shells of “*decussata*” are flat and have a sharply keeled last whorl. The aperture is oval and dark brown inside with a white lip and a strong basal tooth. “*Tingitanaminetteidecussata*” has a network-like sculpture on its surface (Fig. 25). This is in contrast to *Otalatingitana* with a rather smooth surface and a few weakly developed radial ribs. In this species, the interior of the aperture is brighter and the basal tooth conspicuously smaller. Typically, *O.xanthodon* has a smooth shell with evenly rounded whorls and up to three apertural denticles.

**Figure 24.**
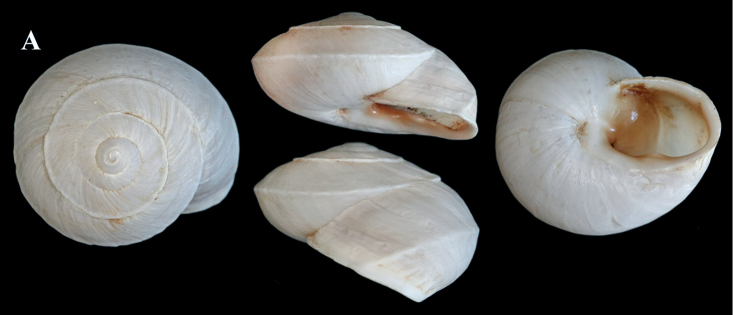
*Otalatingitana* (NMBE 510549), Tarzout de Guigou, Morocco, D = 27.42 mm, H = 14.38 mm (specimens from the type locality of *Archelixminettei* Pallary, 1917). All photographs by Kneubühler, shell × 1.5.

**Figure 25.**
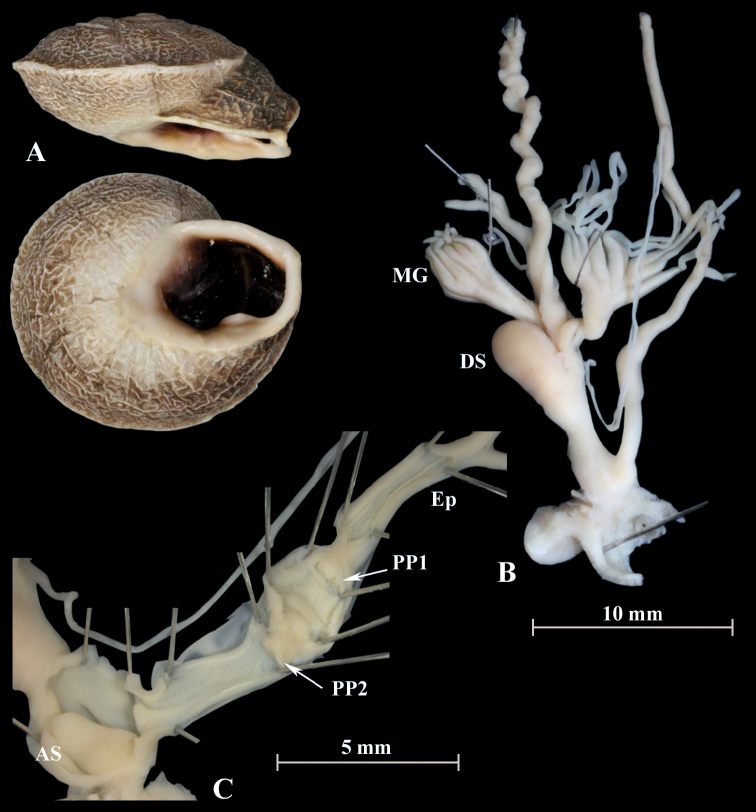
“*Tingitanaminetteidecussata*” (NMBE 549840), Kebdana, Morocco; **A** shell **B** situs **C** atrium and penis; D = 32.45 mm, H = 16.12 mm, situs length 28.84 mm (atrium-flagellum). Situs not complete. All photographs by Kneubühler, shell × 1.5.

The genital organs of “*decussata*” are characterised by two distinct penial papillae, each with a central pore. The distal penial lumen between the atrial stimulator and the distal penial papilla (PP2) is characterised by a network of irregularly shaped folds with large and small ridges. The penial chamber exhibits many annular tissue folds and is ca. 3 mm long. Between the proximal penial papilla (PP1) and the epiphallus is a short transformation zone. The epiphallus is characterised by two strong and several smooth longitudinal ridges. The mucus glands consist of two massive stems which subdivide into several thinner branches which again become thinner in the second half. The dominant structure in the atrium is a large tongue-shaped stimulator. There are almost no differences in the inner and outer morphology of the genital organs of “*decussata*” and *O.xanthodon* specimens.

**Remarks**. According to field observations by R Hutterer, this particular taxon does only occur on top of one mountain in the Kebdana range; comparison with similar specimens illustrated by Cossignani (2014: 109) from Ras el Ma and Tazouta is pending. The distribution area of *O.tingitana/minettei* is far and separated by lowlands, so a position of this taxon as a species in its own right is highly probable. However, as long as topotypic specimens of *O.tingitana* are missing in the genetic analysis, the exact taxonomic position of “*decussata* Pallary” remains unclear. Our results signal a position within or close to *O.xanthodon* rather than to *O.tingitana*.

## Discussion

The results of our study strongly support the monophyly of the genera *Alabastrina* and *Otala* within the tribe Otalini. *Alabastrinaalabastrites* is morphologically as well as genetically clearly separated from the genera *Siretia* and *Otala*. All investigated specimens within *Alabastrina* show the unique trait of the presence of a blind penial appendix. This is an anatomical character, which has never been reported before within the Helicidae. The function of this penial appendix is not known. Schileyko’s system which was based on morphology only, is incorrect as we could demonstrate in our phylogeny that the species *Archelixpallaryi* Kobelt, 1909, which is the type species for the genus *Siretia*, clusters outside the *Alabastrina* clade. We consider this taxon as a separate genus. Anatomical and genetic data for *Helixbailloni* Kobelt, 1888, the type species of *Guilia* Pallary, 1926 also suggest a phylogenetically separate position of this genus (Kneubühler et al. in prep.). The position of *A.tistutensis* within the clade of *A.alabastrites* shows that this extreme local shell form should probably be considered a local subspecies rather than a species in its own rights. Further sampling is necessary to resolve the problem.

The phylogenetic results clearly show that *Siretia* is separated from *Alabastrina*. In the ML analyses *Siretia* forms a lineage separate from *Otala* (Fig. 2; Suppl. materials 1, 2). However, in the Bayesian Inference analyses, *Siretia* clusters within the *Otala* clade (Fig. 3; Suppl. materials 3, 4). Thus, the monophyly of *Otala* is not supported. It cannot be excluded that Siretia forms a subgenus or even a synonym of *Otala*. Unfortunately, we cannot present anatomical data for *S.pallaryi* because of the bad preservation of the only specimen we could analyse. More sequence data are necessary to corroborate the monophyly of *Otala* and to resolve the relationships within the *Otala* clade (including *Siretia*). For the time being, *Siretia* is considered here as a separate unit because of the differences in shell shape. Holyoak and Holyoak (2017: 423) regard *Siretia* as a distinct genus within the Otalini.

*Otalalactea* is characterized by a dark aperture, which clearly differentiates it from *O.punctata* with a white aperture. We investigated several populations of *O.lactea* from Morocco, Spain and Portugal and they all cluster together in the phylogenetic analysis. Hesse’s (1911) investigations of the outer morphology of the genital organs of *Archelixpunctata*, *A.lactea*, and *A.lucasi* showed no difference to our results. In contrast to Holyoak and Holyoak (2017), we could distinguish the species *O.lactea* and *O.punctata* without any doubt by their genital anatomy. *Otalapunctata* has one strongly developed penial papilla and a second which is nearly completely reduced, whereas *O.lactea* has two massive and distinct penial papillae. Unfortunately, Holyoak and Holyoak (2017: 425, Table 1) do not describe the form of the proximal verge (PP1 herein) for each species nor do they provide a drawing. This hampers the interpretation of the data known so far and we agree that more detailed study may be necessary for a reliable comparison of species.

We also investigated specimens of *O.l.murcica* from Almería, Spain; from a genetic point of view there is no difference to the remaining specimens of *O.l.lactea*. The two specimens of *O.l.murcica* included in the analyses from the same population (NMBE-554175 and NMBE-554176 in Figs 2, 3) cluster together with the Portuguese specimen of *O.lactea*, which originates close to the type locality of the neotype of *O.lactea* designated by Holyoak and Holyoak (2017: 446). For this reason we conclude that this subspecies has to be considered a synonym of *O.lactea*.

The specimen from Etsedda, Morocco (NMBE-545594 in Figs 2, 3) clusters as the sister lineage of all investigated *O.lactea* specimens. It shows a slightly different shell morphology and genital anatomy (Fig. 16A, B, C). The shells of this population strongly resemble *Helixlucasii* (syntype shown under Fig. 16D). However, the bootstrap support value for this clade (65) is too low to currently allow the separation as a distinct species or whether it falls within the range of variability of *O.lactea*. More specimens are needed here to corroborate the differences in the anatomical details of the genital organs as well as the separate position on the phylogeny.

“*Tingitanaminetteidecussata*” clusters within the specimens of *O.xanthodon* but with a low support (Figs 2, 3). The genital organs show strong similarities to other *O.xanthodon* specimens as exemplified by the system of two penial papillae, the short penial chamber, the massive mucus glands, and the large atrial stimulator. However, the shell morphology of this form is clearly different. This could be due to a local adaptation to a rocky habitat since the gastropod shell form is strongly influenced by the substrate the species live on (Goodfriend 1986); specimens with a flat shell can hide more easily in crevices, particularly in limestone. This conflicts with the definition of *Tingitana* by Pallary, who erected this genus for species with a keeled shell. Next to the observation cited above that keeled shells are probably an adaptation to a rocky environment with crevices, juvenile shells of large helicid species often show this phenomenon of a keeled shell (see for example species of *Levantina* Kobelt, 1871, *Codringtonia* Kobelt, 1898, *Isaurica* Kobelt, 1901, etc. (Holyoak and Holyoak 2017)). Consequently, this trait is unsuitable for generic definition; its use even for species delimitation is disputable.

Holyoak and Holyoak (2017) synonymised *H.zaffarina* (a species usually under *Dupotetia*) with *O.xanthodon*. Therefore, we included a specimen that usually would have been identified as *D.zaffarina* in our study (Fig. 23A), and compared the shell with that of the syntype (Fig. 23D). We agree here with the synonymisation of *H.zaffarina* with *O.xanthodon*, because our genetic analyses revealed that this specimen clusters within the specimens of *O.xanthodon*.

## Supplementary materials

Supplementary material 1 Maximum Likelihood (RAxML) tree based on mitochondrial data set of COI and 16S Data type: PDF file Explanation note: Numbers represent bootstrap support values from the ML analysis. This dataset is made available under the Open Database License (http://opendatacommons.org/licenses/odbl/1.0/). The Open Database License (ODbL) is a license agreement intended to allow users to freely share, modify, and use this Dataset while maintaining this same freedom for others, provided that the original source and author(s) are credited.Jeannette Kneubühler, Rainer Hutterer, Beat Pfarrer, Eike Neubert

Supplementary material 2 Maximum Likelihood (RAxML) tree based on nuclear data set of H3 and 5.8 S rRNA+ITS2 Data type: PDF file Explanation note: Numbers represent bootstrap support values from the ML analysis. This dataset is made available under the Open Database License (http://opendatacommons.org/licenses/odbl/1.0/). The Open Database License (ODbL) is a license agreement intended to allow users to freely share, modify, and use this Dataset while maintaining this same freedom for others, provided that the original source and author(s) are credited.Jeannette Kneubühler, Rainer Hutterer, Beat Pfarrer, Eike Neubert

Supplementary material 3 Bayesian Inference tree based on mitochondrial data set of COI and 16S Data type: PDF file Explanation note: Numbers represent Bayesian posterior probabilities. This dataset is made available under the Open Database License (http://opendatacommons.org/licenses/odbl/1.0/). The Open Database License (ODbL) is a license agreement intended to allow users to freely share, modify, and use this Dataset while maintaining this same freedom for others, provided that the original source and author(s) are credited.Jeannette Kneubühler, Rainer Hutterer, Beat Pfarrer, Eike Neubert

Supplementary material 4 Bayesian Inference tree based on nuclear data set of H3 and 5.8 S rRNA+ITS2 Data type: PDF file Explanation note: Numbers represent Bayesian posterior probabilities. This dataset is made available under the Open Database License (http://opendatacommons.org/licenses/odbl/1.0/). The Open Database License (ODbL) is a license agreement intended to allow users to freely share, modify, and use this Dataset while maintaining this same freedom for others, provided that the original source and author(s) are credited.Jeannette Kneubühler, Rainer Hutterer, Beat Pfarrer, Eike Neubert
