# Supplementary figures and images for: Anatomical and phylogenetic investigation of the genera Alabastrina Kobelt, 1904, Siretia Pallary, 1926, and Otala Schumacher, 1817 (Stylommatophora, Helicidae)
Source: Zookeys. 2019 May 9;843:1–37. doi: 10.3897/zookeys.843.32867 (PMC6522470; doi:10.3897/zookeys.843.32867)

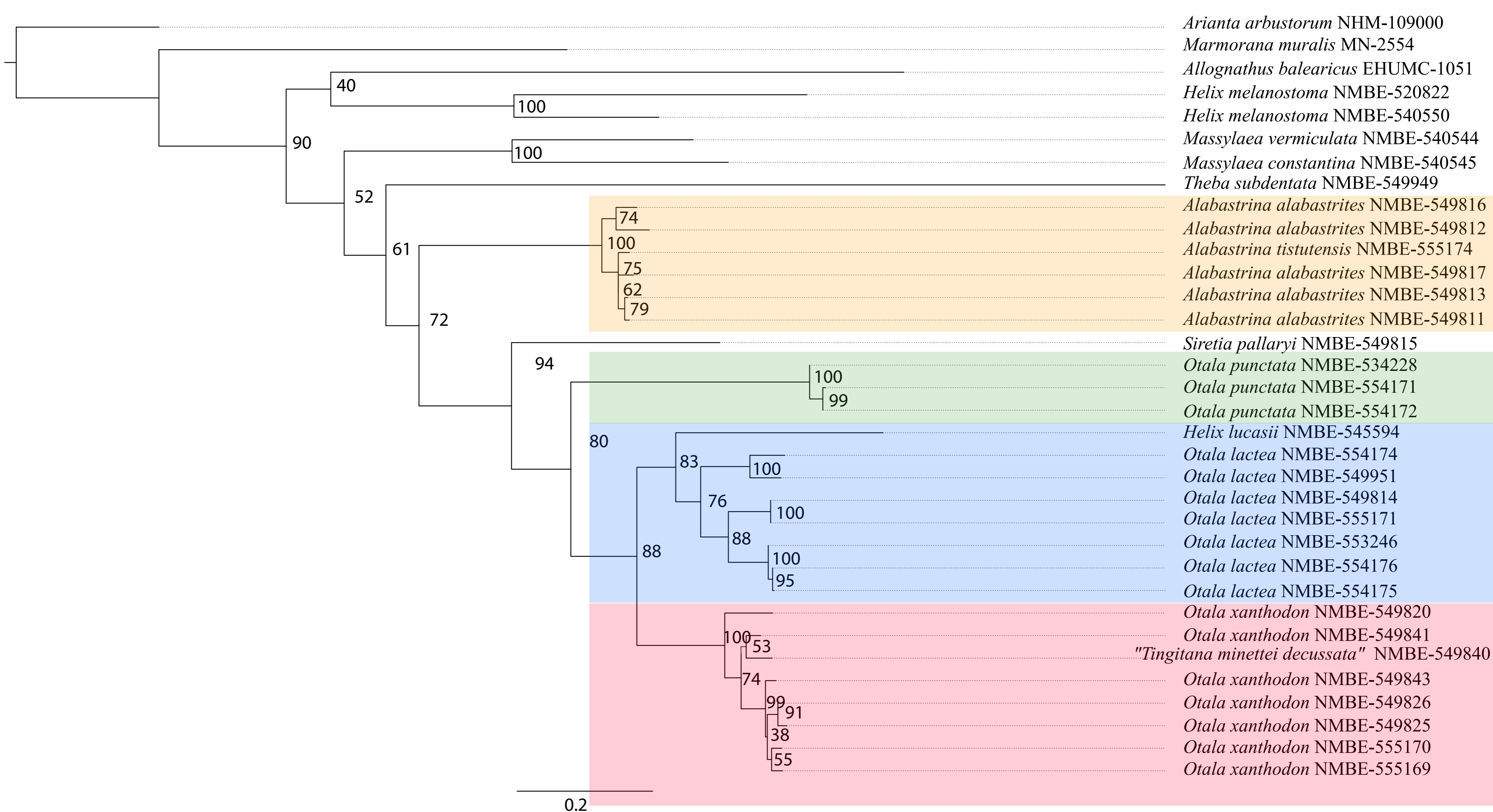

Supplement: Supplementary material 1 [file zookeys-843-001-s001.pdf]

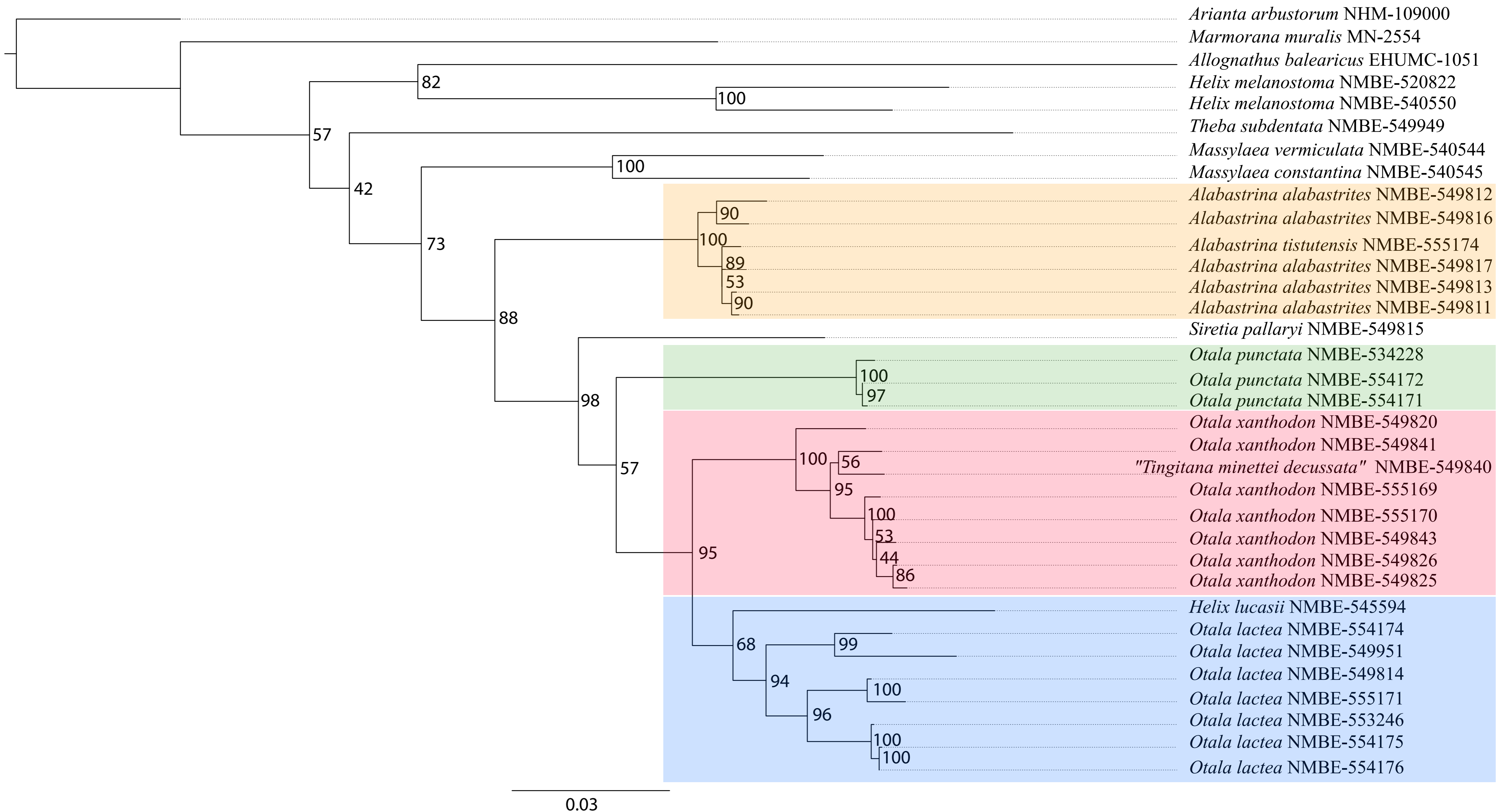

Supplement: Supplementary material 2 [file zookeys-843-001-s002.pdf]

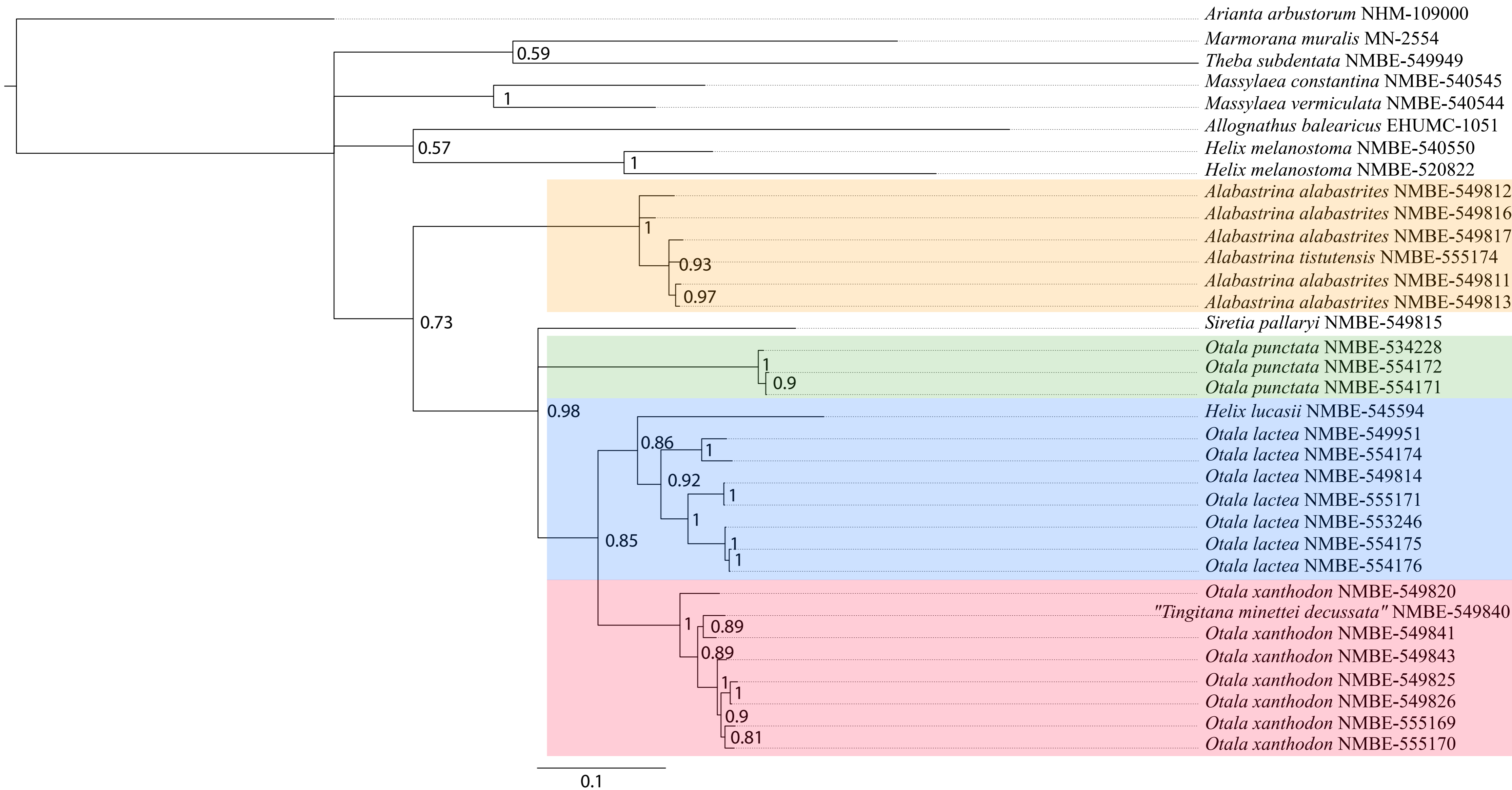

Supplement: Supplementary material 3 [file zookeys-843-001-s003.pdf]

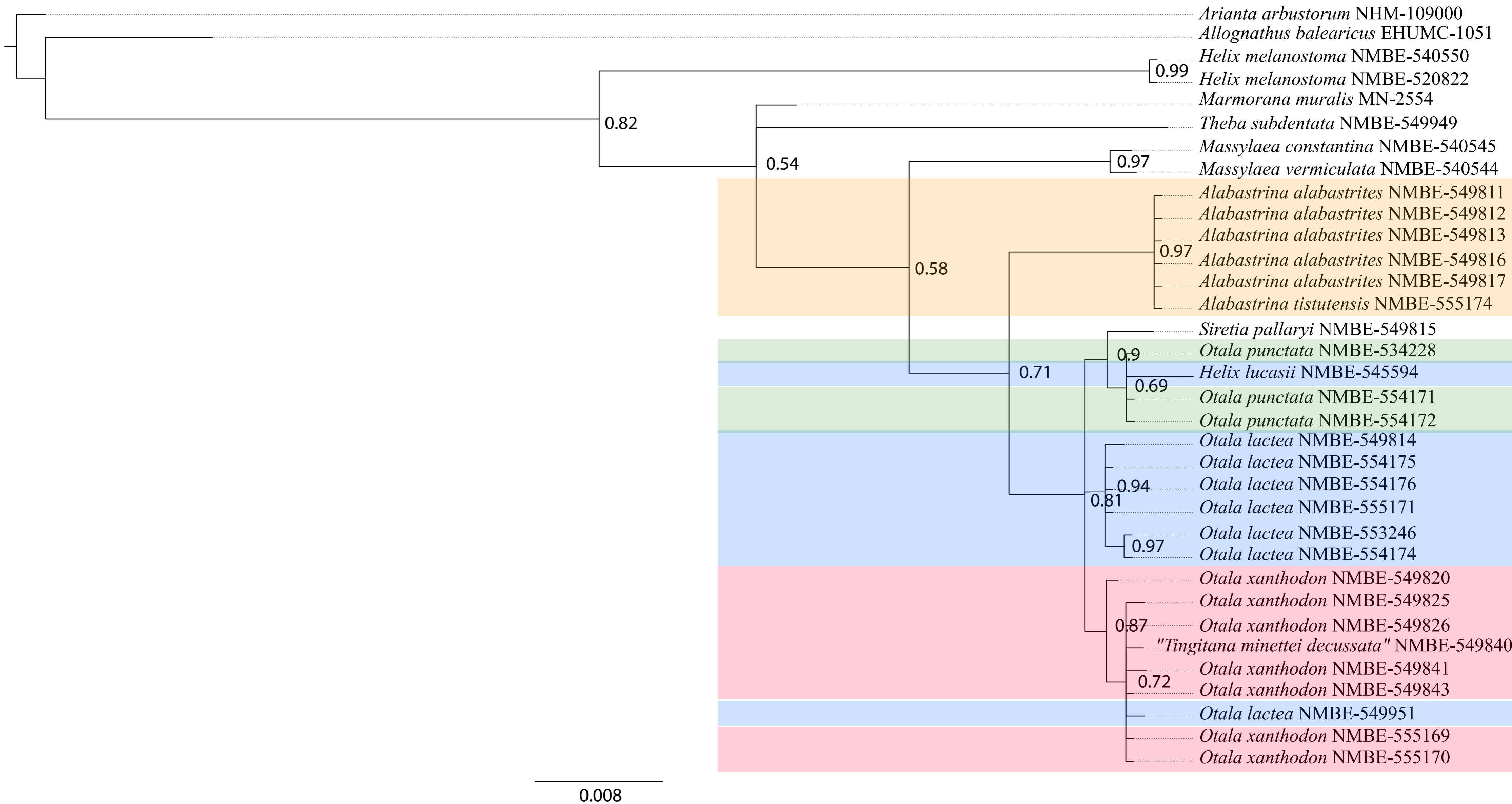

Supplement: Supplementary material 4 [file zookeys-843-001-s004.pdf]
